# Systematic elucidation of the cross-omics regulatory network in chronic rhinosinusitis: the LAT-IL23R metabolic axis

**DOI:** 10.1016/j.bjorl.2026.101819

**Published:** 2026-04-22

**Authors:** Zongxiong Ou, Chen Gao

**Affiliations:** a Clinical Oncology School of Fujian Medical University, Fujian Cancer Hospital, Head and Neck Surgeons, Fujian, China; b The First Hospital of Putian City, Department of Otorhinolaryngology, Putian, Fujian, China

**Keywords:** Chronic rhinosinusitis, LAT-IL23R metabolic axis, Protein-protein interaction network, Mediation analysis, Mendelian randomization

## Abstract

**Objective:**

The pathological mechanisms underlying Chronic Rhinosinusitis (CRS) remain incompletely understood, and current treatments exhibit low response rates alongside a scarcity of specific drugs targeting the disease’s fundamental characteristics. Although dysregulation of protein interactions and metabolic reprogramming have been confirmed as contributors to the progression of CRS, a systematic elucidation of the cross-omics regulatory network encompassing genetics, proteins, and metabolites is still needed.

**Methods:**

This study employed a multi-stage comprehensive analytical strategy that integrates MR, mediation analysis, and proteomics. Initially, using pQTL data from the deCODE and UKB-PP databases, as well as CRS GWAS data from the FinnGen database, MR was utilized to identify proteins associated with CRS. Following this, a two-step mediation analysis was conducted to construct a protein-protein-metabolic regulatory network.

**Results:**

The study identified that the LAT-IL23R metabolic axis mediates CRS through γ-glutamyltyrosine and trans-4-hydroxyproline.

**Conclusion:**

This study systematically reveals, for the first time, the promoting role of the LAT-IL23R-amino acid metabolic network in CRS, providing a theoretical basis for the development of targeted combination therapies.

**Level of Evidence:**

Level 3 (causal inference from cohort-derived genetic data).

## Highlights

• Systematic elucidation of LAT-IL23R amino acid metabolic network in CRS.
• Construction of “gene-protein-metabolite” regulatory network via MR and mediation.
• LAT-IL23R axis modulates CRS via γ-glutamyltyrosine and trans-4-hydroxyproline.
• Theoretical basis for targeted therapies against chronic rhinosinusitis.

## Introduction

Chronic Rhinosinusitis (CRS) is a chronic inflammatory disease that affects the nasal and sinus mucosa, characterized by a complex and not fully elucidated pathogenesis. While environmental factors and microbial infections are recognized as key triggers, significant gaps remain in our understanding of their core molecular mechanisms. Most patients exhibit limited responses to existing pharmacological therapies, and there are currently no disease-modifying agents that target the core pathological processes.^1^ Recent investigations have revealed that the dysregulation of protein interaction networks and imbalances in metabolites may drive the sustained inflammatory progression in CRS.^2^^,^^3^ These regulatory disturbances lead to the disruption of the mucosal barrier, amplified inflammatory signaling, and tissue fibrosis; however, the precise molecular regulatory networks remain undefined. This knowledge gap directly impedes the development of precision therapeutic strategies.

The dynamic dysregulation of protein networks serves as a central driver of CRS pathology. This dysregulation influences disease progression by modulating inflammatory signaling, maintaining epithelial barrier integrity, and facilitating tissue repair mechanisms.^4^ In CRS, the aberrant activation of immune-related protein networks triggers the uncontrolled release of proinflammatory cytokines and chemokines, leading to exaggerated local immune responses and compromised anti-inflammatory regulation.^5^ Furthermore, protein dysfunction disrupts critical mucosal barrier structures, thereby increasing the risk of pathogen invasion and perpetuating inflammatory cycles.^6^ Additionally, the reprogramming of tissue repair-associated proteins promotes abnormal fibroblast activation and excessive extracellular matrix deposition, contributing to irreversible sinus fibrosis.^7^ Importantly, while current therapies provide transient suppression of partial inflammatory responses, they do not systematically correct global protein network imbalances or restore metabolic-immune homeostasis.

The pathogenesis of CRS is intricately associated with disturbances in the metabolic regulatory network. Within the CRS microenvironment, the abnormal accumulation of reactive oxygen species, lipid mediators, and metabolic reprogramming intensifies oxidative stress and inflammatory signaling, resulting in immune cell dysfunction and tissue damage.^8^^,^^9^ Immunometabolic dysregulation is particularly pronounced in specific immune subtypes, where the synergistic activation of glycolytic pathways perpetuates proinflammatory phenotypes. Concurrently, alterations in nasal microbiota composition disrupt host energy metabolism through metabolite interactions, such as imbalances in short-chain fatty acids, thereby exacerbating epithelial barrier dysfunction.^10^ These metabolic abnormalities underscore the therapeutic potential of targeting metabolic reprogramming, although current interventions are still limited in their ability to achieve comprehensive metabolic-immune regulation.

The integration of Mendelian Randomization (MR) and mediation analysis offers unique advantages for dissecting protein interaction networks and metabolic regulatory mechanisms. MR employs genetic variants as instrumental variables to establish causal relationships between specific proteins and diseases, effectively addressing confounding biases and reverse causality inherent in observational studies.^11^ Building on this, mediator analysis further delineates causal pathways by distinguishing direct protein effects from indirect effects mediated through intermediate proteins or metabolites. This combined strategy is particularly powerful for investigating dynamic alterations in protein interaction and metabolic networks, enabling the identification of both direct and indirect causal pathways while pinpointing critical mediator proteins and key metabolites.^12^^,^^13^ Through this approach, researchers gain deeper insights into disease pathogenesis and identify novel therapeutic targets for precision medicine. Collectively, the integration of MR and mediator analysis provides a robust framework for unraveling the ‘gene-protein-phenotype’ cascade, offering critical insights into the complex biological mechanisms underlying diseases.

Through a multi-stage integrative analysis, this study systematically investigated the potential genetic regulatory mechanisms underlying the protein-protein-metabolic networks in Chronic Rhinosinusitis (CRS). Proteins significantly associated with CRS risk were initially identified using protein Quantitative Trait Loci (pQTL) data from the Icelandic deCODE cohort and Genome-Wide Association Study (GWAS) data from the Finnish FinnGen database. Subsequent integration of pQTL data from the UK Biobank with FinnGen GWAS results allowed for the inference of upstream regulatory factors. A two-step mediator analysis revealed a critical metabolite-associated protein regulatory axis, namely LAT-IL23R. Further analysis suggested that IL23R may modulate disease progression through downstream metabolites such as gamma-glutamyltyrosine and trans-4-hydroxyproline. This study provides the first theoretical evidence indicating a pivotal role of the LAT-IL23R-amino acid metabolic network in the pathogenesis of CRS. The established multi-dimensional “gene-protein-metabolic regulatory network” offers insights into its potential molecular mechanisms and lays a theoretical foundation for future development of targeted precision therapies.

## Methods

### Study design

**Fig. 1:**
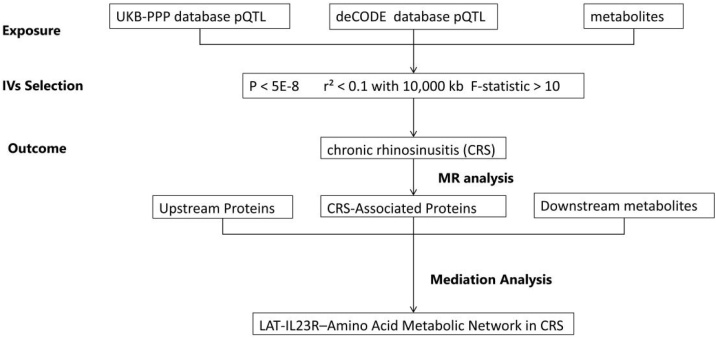
Study workflow. MR, Mendelian Randomization; pQTL, protein Quantitative Trait Locus.

Fig. 1 illustrates the overall workflow of this study. We utilized pQTL data from the deCODE and UKB-PP databases as exposures, and GWAS data for CRS from the FinnGen database as outcomes to conduct a two-sample MR analysis. The aim was to investigate causal relationships between proteins and CRS. Subsequently, we performed mediator analysis to identify potential molecular regulatory axes and explore downstream mechanisms, ultimately constructing a genetic regulatory network of protein-protein-amino acid metabolism. During the analysis, Single Nucleotide Polymorphisms (SNPs) that met strict inclusion and exclusion criteria were selected as Instrumental Variables (IVs). A series of sensitivity analyses were conducted to ensure the reliability of the MR results. All analyses adhered to rigorous ethical standards, with data obtained from original studies that had received ethical approval and participant informed consent.

### Data sources

Proteomic data were obtained from two primary sources, both of European ancestry: (1) The pQTL data of the Icelandic population available in the deCODE database (https://www.decode.com/summarydata/)^14^; and (2) Plasma proteomics pQTL data from the UK Biobank Pharma Proteomics Project (UKB-PPP; https://www.synapse.org/Synapse:syn51364943/wiki/622119).^15^

Metabolite data were derived from the Canadian Longitudinal Study on Aging (CLSA) cohort, encompassing 1,091 individual metabolites and 309 metabolite ratios measured in 8,299 participants. Detailed chemical characteristics of the 1,400 plasma metabolites are presented in Table S1.^16^

The CRS GWAS summary statistics (FinnGen J10_CHRONICSINUSITIS phenotype, Release R12) were sourced from FinnGen. The phenotype is defined by ICD-10 code J32 (Chronic sinusitis) and includes both CRSwNP and CRSsNP subtypes; however, subtype stratification data are not available. The dataset comprises 22,099 CRS cases and 371,520 controls. We used FinnGen’s pre-adjusted summary statistics, as FinnGen had adjusted for age, sex, and population stratification according to its standardized pipeline. This adjustment ensures that the outcome data used in our analyses are controlled for age and sex confounding factors, thereby supporting reliable causal inference.

All four datasets comprise individuals of European ancestry. Additionally, the absence of sample overlap between UKB-PPP and FinnGen has been confirmed through official cohort documentation and database usage agreements. Proteomic measurements (deCODE, UKB-PPP) and metabolomic measurements (CLSA) were performed on plasma samples.

### Instrumental Variables (IVs)

The selection of Single Nucleotide Polymorphisms (SNPs) adhered to the assumptions of MR Initially, SNPs that achieved genome-wide significance (p < 5.0 × 10^−8^) were retained. Subsequently, independent SNPs were identified utilizing the 1000 Genomes European reference data, applying a Linkage Disequilibrium (LD) threshold of r^2^ < 0.1 and a clumping window of 10,000 κb. Furthermore, SNPs exhibiting incompatible alleles between the exposure and outcome were excluded. Palindromic SNPs were addressed using allele frequency data; those without frequency information were excluded from consideration. Lastly, SNPs with F-statistics <10 were removed to mitigate the risk of weak instrument bias.

### Instrumental Variables (IVs)

The selection of Single Nucleotide Polymorphisms (SNPs) was conducted in strict accordance with the principles of MR.^17^ Initially, we retained SNPs that achieved genome-wide significance, defined as a p-value of less than 5.0 × 10^−8^. Following this, we performed a Linkage Disequilibrium (LD) analysis using the 1000 Genomes European reference data.^18^ We set a clumping window of 10,000 κb and an LD threshold of r^2^ < 0.1 to identify independent SNPs. Additionally, we included SNPs with a Minor Allele Frequency (MAF) greater than 0.01 to avoid low-frequency variants, which could introduce sampling errors. For palindromic SNPs, specifically those categorized as A/T or C/G, we excluded any that lacked allele frequency information. For those with available frequency data, we ensured that the allele directions were aligned between the exposure and outcome datasets, confirming consistency in the reference strand. We also excluded SNPs with incompatible alleles between the two datasets, as well as palindromic SNPs without frequency information. Lastly, we removed SNPs with F-statistics less than 10 to reduce the potential for weak instrument bias.^19^

### Mendelian randomization, sensitivity, and directionality analyses

Two-sample Mendelian Randomization (MR) analyses were conducted using the TwoSampleMR package, with Inverse Variance Weighted (IVW) regression serving as the primary analytical method.^20^ Exposures that did not yield valid IVW estimates were excluded from the analysis. To enhance the robustness of causal inferences, the IVW results were complemented by additional methods, including MR-Egger, weighted median, simple mode, and weighted mode estimators. Sensitivity analyses were performed to assess the reliability of the findings. This included Cochran’s *Q* test to measure heterogeneity,^21^ MR-Egger intercept tests to evaluate horizontal pleiotropy,^22^ and MR-PRESSO (Mendelian Randomization Pleiotropy RESidual Sum and Outlier) for outlier correction to identify and address pleiotropy caused by outlier SNPs.^23^ Additionally, Steiger tests were utilized to confirm the directionality of causal effects and reduce the risk of reverse causality,^24^ while leave-one-out sensitivity analyses were conducted to ensure that no single instrumental variable SNP disproportionately affected the overall causal effect estimate.

### Mediation analysis

**Fig. 2:**
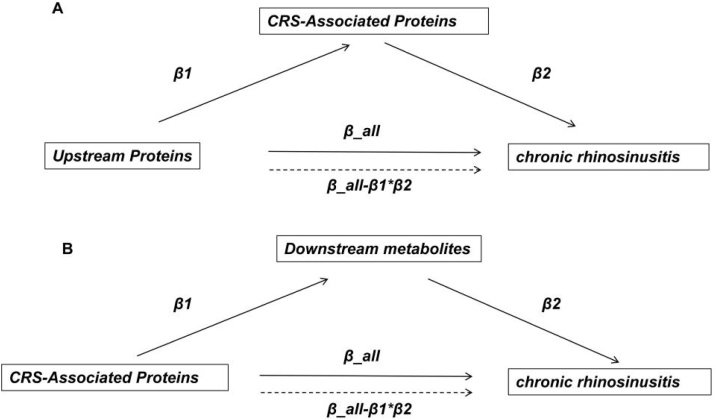
Workflow of mediation analysis for regulatory axis screening.

For the upstream analysis, a two-step MR approach was employed^25^: (1) Estimating the total effect of upstream proteins on CRS (β_all); (2) Calculating the effect of upstream proteins on downstream proteins (β₁) and the effect of downstream proteins on CRS (β₂). The mediation effect (β₁₂) was defined as β₁×β₂, with the mediation proportion calculated as (β₁₂ / β_all) ×100% (Fig. 2A).

For the downstream analysis, we assessed: (1) The total effect of proteins on CRS (β_all); (2) The effect of proteins on metabolites (β₁) and the effect of metabolites on CRS (β₂). The mediation effect (β₁₂ = β₁ × β₂) and mediation proportion (β₁₂ / β_all ×100%) were calculated (Fig. 2B). The delta method was used to compute Standard Errors (SE), 95% Confidence Intervals (95% CI), and p-values for mediation effects (including β₁₂, the product of β₁ and β₂). Results with p < 0.05 and mediation proportions >15% were prioritized for further investigation ‒ this 15% cutoff is justified by prior methodological studies in multi-omics Mendelian Randomization (MR) mediation, which recommend it as a threshold to identify biologically meaningful mediation effects.^26^

### Statistical methods

All analyses were performed using R software (v4.4.2; https://www.r-project.org/). A statistical significance threshold of p < 0.05 was applied.

## Results

### Identification of CRS-associated molecules

Utilizing MR analysis in conjunction with proteomic Quantitative Trait Loci (pQTLs) sourced from the deCODE database as exposures, we identified proteins that are causally associated with CRS. After conducting thorough sensitivity analyses, we retained 95 molecules that exhibited significant associations with CRS (Table S2).

### Screening of upstream regulators

We subsequently employed MR analysis to identify upstream regulators utilizing protein Quantitative Trait Locus (pQTL) data from the UK Biobank Pharma Proteomics Project (UKB-PPP). This analysis identified 50 molecules that were significantly associated with CRS (Table S3). Further MR analyses revealed additional upstream regulatory molecules associated with the CRS-related proteins identified in the deCODE cohort. After conducting sensitivity validation, we established 564 causally linked protein pairs (Table S4).

### Molecular regulatory axis based on mediation analysis

We employed mediation analysis to calculate the p-value of the mediation effect and the mediation proportion using the delta method, and subsequently screened candidate molecules ‒ all full mediation statistics for the tested upstream and downstream axes (including β₁ [effect of exposure on mediator], β₂ [effect of mediator on outcome], β₁₂ [mediation effect], Standard Error [SE] of β₁₂, 95% CI of β₁₂, and p-value of the mediation effect) are provided in Table S5. Through this analysis, we identified 38 molecular pairs with significant mediation relationships, and further selected those with statistically significant and robust effects for follow-up investigation. Concurrently, during screening, we innovatively adopted a combined upstream and downstream mechanism analysis strategy to exclude protein pairs lacking downstream regulatory mechanisms. Ultimately, we inferred a potential LAT-IL23R association axis with significant statistical mediation effects (Fig. 2, Table 1, Table 2, Table 3).

**Table 1:** Causal relationships of CRS ‒ associated proteins and upstream/downstream regulators with CRS identified by mendelian randomization analysis.

Exposure	Outcome	Mendelian randomization analysis				
		Method	p	β	OR	OR (95% CI)
deCODE	FinnGen					
IL23R	CRS	IVW	5.13e-05	0.113	1.119	(1.06, 1.18)
UKB-PPP	FinnGen					
LAT	CRS	IVW	0.046	0.131	1.140	(1.01, 1.29)
UKB-PPP	deCODE					
LAT	IL23R	IVW	8.84e-05	0.208	1.231	(1.11, 1.37)

**Table 2:** Sensitivity analysis and directionality test of causal relationships in mendelian randomization for CRS ‒ associated proteins and upstream/downstream regulators.

Exposure	Outcome	SNP	Steiger direction	Steiger p-value	Heterogeneity	Pleiotropy
deCODE	FinnGen					
IL23R	CRS	rs71080535	TRUE	4.24e-112	0.761	0.291
UKB-PPP	FinnGen					
LAT	CRS	rs892090	TRUE	2.74e-19	0.103	0.790
UKB-PPP	deCODE					
LAT	IL23R	rs6993770	TRUE	4.54e-09	0.146	0.366

**Table 3:** Mediation Effect of the LAT-IL23R–amino acid metabolic network in CRS.

Exposure	Mediator	Proportion mediated				Proportion mediated (95% CI)	p-value
		β_all	β1	β2	β12/β_all		
LAT	IL23R	0.131	0.208	0.113	17.9%	(0.8%, 35%)	0.040
IL23R	Gamma-glutamyltyrosine	0.113	0.161	0.198	28.2%	(5.7%, 50.7%)	0.014
IL23R	Trans-4-hydroxyproline	0.113	0.179	0.142	22.5%	(0.7%, 44.3%)	0.043

### Mediation analysis explores downstream mechanisms of molecular regulatory axis

**Fig. 3:**
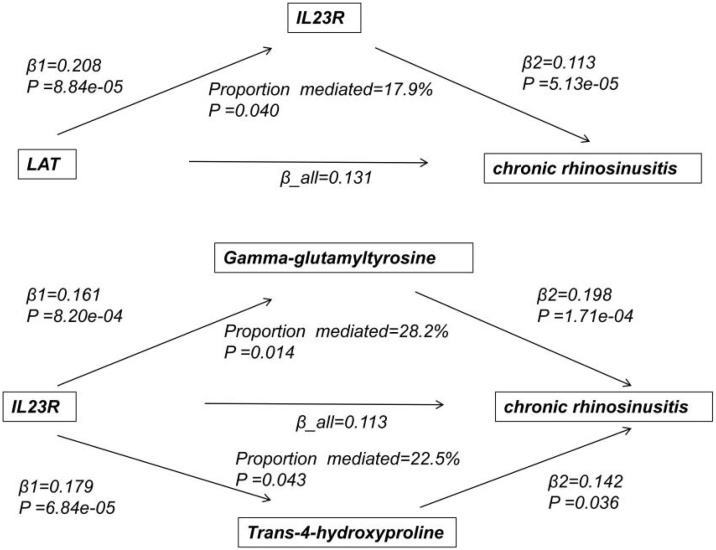
Directed Acyclic Graph (DAG) of mediation analysis for the LAT-IL23R-amino acid metabolic axis in Chronic Rhinosinusitis (CRS).

To investigate the downstream mechanisms of the LAT-IL23R regulatory axis, we conducted mediation analysis by calculating the proportion of metabolites mediating the relationship between proteins and chronic sinusitis (Table S5). Firstly, we employed the MR method to identify metabolites associated with chronic sinusitis (Table S6). Subsequently, we calculated the relationship between proteins and these metabolites using the MR method (Table S6, Table 1, Table 2). Finally, by integrating the findings related to proteins and chronic sinusitis, we identified two amino acid metabolites and constructed a LAT-IL23R-amino acid metabolism network. We assessed the extent of the causal effect of protein-mediated metabolites on chronic sinusitis, as shown in Table 3, Fig. 3 and Figure S3. To comprehensively validate the LAT-IL23R-amino acid axis, we provide detailed data that includes Mendelian randomization results, outcomes from sensitivity analyses addressing pleiotropy, heterogeneity, and directionality verification, as well as MR-PRESSO test results, all of which can be found in Table S8. Information regarding the instrumental variable Single Nucleotide Polymorphisms (SNPs) that play a role in this regulatory axis is presented in Table S7. Furthermore, visual representations of the key validation results are illustrated in Figure S1 and Figure S2, which serve to further confirm the reliability of the LAT-IL23R regulatory axis.

## Discussion

This study systematically explored the genetic associations of the LAT-IL23R-amino acid metabolic network in CRS through multi-stage integrative analyses, and all inferred ‘regulatory relationships’ are based on cross-omics statistical causal models (MR and mediation analysis) rather than direct molecular experimental evidence. The multidimensional evidence chain of the ‘gene-protein-metabolic network’ provides estimates of genetic associations and mediation effects, with the putative pathogenic role and molecular mechanisms requiring further experimental validation to support its potential value for targeted precision therapy research.

Protein-Protein Interaction (PPI) networks, as core regulatory systems for cellular metabolism and signal transduction, exhibit multidimensional regulatory features in CRS pathogenesis. Research indicates that dysregulation of PPI network homeostasis affects metabolic reprogramming, immune response modulation, and tissue remodeling processes, forming the molecular basis of CRS development.^27^ Specifically, this network integrates stress signals and inflammatory mediators, creating cascade amplification effects in phenotypic transformation, matrix deposition, and immune microenvironment regulation. Its dysregulated state may underlie the heterogeneous clinical manifestations of CRS.^28^ From a metabolic perspective, PPI networks act as hubs mediating synergistic or antagonistic interactions between key signaling pathways in energy metabolism and oxidative stress.^29^ Such regulation operates not only at the molecular switch level of epithelial cell phenotypic transformation but also in maintaining metabolic homeostasis during tissue fibrosis. Notably, PPI networks influence spatiotemporal release patterns of inflammatory mediators by bridging innate and adaptive immune interactions, making them critical molecular carriers for Th cell differentiation and polarization.^30^ Systems biology studies further suggest that CRS-associated PPI networks exhibit tissue specificity and disease-stage dependency ‒ interaction modules within specific cell subsets dynamically reorganize under microenvironmental stress.^31^ These topological changes may explain disease chronicity and drug resistance. Establishing multi-scale analytical frameworks to elucidate interactions between network regulatory advantages and compensatory pathways will help uncover core pathological mechanisms and guide precision therapeutic strategies.

Gamma-glutamyltyrosine and trans-4-hydroxyproline, two bioactive metabolites with distinct properties, may play differential roles in the pathophysiology of CRS. Elevated levels of gamma-glutamyltyrosine, a glutathione-derived metabolite catalyzed by glutamyltransferase, correlate with oxidative stress and inflammation, potentially exacerbating tissue damage through immune cell infiltration and the release of pro-inflammatory cytokines.^32^ Notably, in CRS patients, this metabolite may activate the TLR4/NF-κB pathway, promoting the secretion of IL-6 and IL-8, thereby aggravating mucosal inflammation and remodeling;^33^ the potential involvement of mast cells in this process remains hypothetical and requires further validation. In contrast, trans-4-hydroxyproline, a collagen-specific modified amino acid, primarily regulates Extracellular Matrix (ECM) homeostasis, with elevated levels directly linked to fibrosis in CRS.^34^^,^^35^ These metabolites synergize through multiple mechanisms: pathologically, gamma-glutamyltyrosine disrupts mucosal barrier integrity via oxidative stress, creating a microenvironment conducive to trans-4-hydroxyproline-driven fibrosis and perpetuating an imbalance between damage and repair. Immunologically, they induce polarization of Th cell subsets through distinct pathways κ-gamma-glutamyltyrosine suppresses specific pro-inflammatory mediators, while trans-4-hydroxyproline enhances effector cell recruitment, collectively exacerbating inflammatory heterogeneity.^36^ Therapeutically, targeting these metabolites offers complementary strategies: enhancing antioxidant defenses may mitigate the effects of gamma-glutamyltyrosine, whereas restoring collagen metabolism homeostasis could block trans-4-hydroxyproline-driven fibrosis. The combined detection of both metabolites improves the accuracy of disease subtyping their dynamic changes may reflect treatment responses, though this potential as a therapeutic monitoring indicator awaits clinical confirmation, offering novel intervention targets and laying the groundwork for precision medicine in CRS.

Our study estimates that the LAT-IL-23R-amino acid metabolic functional network is associated with inflammatory cascades and tissue remodeling in CRS, with its role as a potential driver requiring further functional confirmation. Specifically, LAT and IL-23R, as critical immune regulators, jointly promote CRS pathogenesis through metabolic reprogramming. LAT, a signaling hub in immune cells, modulates lymphocyte activation thresholds and differentiation by engaging key intermediary signaling pathways^37^ ‒ including STAT3, NFAT, and potentially NF-Kb ‒ that are known to regulate IL-23R expression.^38^^,^^39^ Dysfunction of LAT leads to aberrant pro-inflammatory cytokine release. IL-23R, a membrane receptor that sustains chronic inflammation, bridges innate and adaptive immunity.^40^ Based on relevant studies, we speculate that their hierarchical coupling is associated with metabolic-immune crosstalk in CRS: LAT regulates IL-23R through the interaction of these three pathways: (1) Phosphorylated STAT3 (activated by LAT-driven TCR signals) forms dimers, translocates to the nucleus, and directly binds the IL-23R promoter while recruiting histone acetyltransferase to relax chromatin^41^^,^^42^; (2) LAT-phosphorylated PLCγ1 triggers calcium release, activating calcineurin to dephosphorylate NFAT ‒ nuclear NFAT then binds the IL-23R promoter and forms a “NFATc1·STAT3” complex to synergize transcription^43^^,^^44^; (3) NF-κB indirectly supports IL-23R by inducing IL-23 (which activates STAT3 via paracrine signaling) and acting as an upstream activator of JAK2/STAT3.^45^ These pathways also form a positive feedback loop: STAT3 activates NFATc1 transcription, and NFATc1 enhances STAT3 acetylation, thereby reinforcing their mutual activation. To date, no direct transcriptomic or proteomic data from CRS airway tissues, such as sinus mucosal biopsies, validate this axis. However, LAT’s role in TCR signaling, the IL-23R-Th17 pathway’s involvement in CRS inflammation, and the conserved immune functions of STAT3, NFAT, and NF-κB support the plausibility of this hypothesis. This hypothesis must be confirmed by in vitro and in vivo molecular experiments. As a key organizer of immune synapses, LAT’s expression directly influences the balance of T-helper cell differentiation.^46^^,^^47^ In CRS, LAT hyperactivity disrupts immune homeostasis by activating the IL-23R axis ‒ not through non-specific receptor regulation,^48^ but via coordinated activation of STAT3, NFAT, and NF-κB. LAT amplifies TCR signals to promote STAT3 phosphorylation and NFAT nuclear translocation, while indirectly sustaining an NF-κB-dependent inflammatory microenvironment, collectively enhancing IL-23R transcription in Th17 cells. Conversely, IL-23R signaling remodels immune cell metabolic pathways, enhancing the biosynthesis of gamma-glutamyltyrosine and trans-4-hydroxyproline. Their synergy manifests as a cascade of metabolic-immune regulation. LAT primes immune activation, enabling sustained IL23R signaling in inflamed tissues. Meanwhile, IL23R-driven metabolic reprogramming maintains Th cell pro-inflammatory phenotypes and generates metabolites that further amplify LAT activity.^49^ This dynamic interaction creates a self-perpetuating pathological loop, leading to metabolite accumulation. For example, gamma-glutamyltyrosine sustains epithelial stress by impairing antioxidant defenses, while trans-4-hydroxyproline restructures the Extracellular Matrix (ECM),^50^ consistent with its role in collagen metabolism regulation. Whether this ECM remodeling facilitates immune cell infiltration remains to be verified. Future studies should systematically characterize the spatiotemporal expression of this axis and its multidimensional interactions with microbiomes and epigenetic modifications, as these are critical for developing precision therapies.

The strengths of this study lie in its multi-level, multidimensional analytical strategy. First, we integrated proteomic and metabolomic data through MR and mediation analyses to construct a comprehensive evidence chain of the “gene-protein-metabolic network”, thereby enhancing the robustness of our results. Second, the use of large-scale datasets, including deCODE, UKB-PPP, GWAS Catalog, and FinnGen, ensured broad applicability and representativeness. Third, rigorous sensitivity analyses were conducted to minimize potential biases. Finally, this study characterizes the genetic and metabolic associations of the LAT-IL23R-amino acid network in CRS, providing novel preliminary insights for microenvironment-targeted therapy research, with clinical implications dependent on further functional and translational validation.

However, several limitations of this study should be acknowledged and can be organized into three dimensions. First, concerning data source representativeness, the data primarily originate from European populations. This restricts the generalizability of the findings to other ethnic groups. Therefore, future research should aim to include more diverse populations to verify the consistency of the LAT-IL23R-amino acid metabolic axis across different ethnicities. Moreover, the FinnGen CRS GWAS summary data used in this study lack stratification information for CRS subtypes. This precludes the dissection of their independent associations with the LAT-IL23R-amino acid metabolic axis and limits subtype-specific interpretation of the results. Furthermore, all pQTL and metabolomic data used in this study were derived from plasma samples. Since the pathological core of Chronic Rhinosinusitis (CRS) lies in local sinus mucosal inflammation, plasma-derived protein and metabolite levels may not fully reflect the true expression levels and metabolic profiles in sinus mucosal tissue. This limitation may introduce tissue-specific bias. To address this issue, future studies could incorporate multi-omics data from sinus mucosal biopsies to further validate the regulatory role of the identified axis in local lesions and reduce tissue-specific bias.Second, concerning limitations related to analytical methodology and confounding control, Mendelian Randomization (MR) offers advantages in mitigating certain biases; however, it does not fully account for the intricate interactions between proteins. Although core associations were validated through sensitivity analyses, residual confounding may still affect results due to inherent limitations of GWAS and pQTL data. These include limited consideration of individual-level factors such as renal or hepatic function in the metabolomic analysis. While the two-step MR design, employing genetically specific Instrumental Variables (IVs), may reduce some potential confounding by isolating the genetic effect of metabolites from systemic metabolic status, the influence of non-genetic pathways on plasma metabolite levels cannot be completely ruled out. Finally, the p-values in this study were not adjusted for multiple comparisons, which increases the likelihood of false positives for secondary molecules. Therefore, individual associations should be interpreted within their biological context to avoid overinterpreting spurious links. Moreover, although the two-step MR design and sensitivity analyses helped mitigate potential confounding from related amino acid pathways, we did not explicitly validate the independent regulatory role of the identified metabolites against other key molecules within these pathways. This leaves room for further exploration to clarify the hierarchical relationships between the target metabolites and other pathway components.As for limitations related to mechanism validation and network complexity, additional experimental validation is essential to elucidate the molecular regulatory mechanisms underlying the LAT-IL23R-amino acid metabolic axis, as current causal inferences are based on statistical models and direct experimental evidence for the axis’s role in CRS pathogenesis is still lacking; moreover, the complexity of the LAT-IL23R network extends beyond the scope of this analysis, as this axis may interact with various other pathways and be influenced by factors such as gut microbiota and epigenetics ‒ factors not incorporated in the current analysis ‒ highlighting the need for more comprehensive multi-dimensional investigations in the future.

## ORCID ID

Zongxiong Ou: 0009-0005-2618-9424

## Data availability statement

The analytical approach of this study complies with ethical standards. All data used in this study come from original studies that obtained prior approval from their respective ethics committees, and all participants in the original cohorts signed written informed consent forms. Detailed information on all datasets used in this study is summarized in Table S9, which includes the source, access method, sample characteristics, and acquisition details of each dataset. The datasets are described below.

## Proteome data

- Plasma proteomic Quantitative Trait Locus (pQTL) data for the Icelandic population were obtained from the deCODE database (URL: https://www.decode.com/summarydata/). This dataset is publicly available as fixed content ‒ meaning it does not have a separate version number ‒ and was downloaded for this study in May 2025.

- Plasma proteomic pQTL data from the UK Biobank Pharmaceutical Proteomics Project (UKB-PPP) are accessible at https://www.synapse.org/Synapse:syn51364943/wiki/622119. This dataset is also publicly available as fixed content without a separate version number and was downloaded in May 2025.

2. Metabolite Data: Metabolomic data were obtained from the Canadian Longitudinal Study on Aging (CLSA) cohort (official website: https://www.clsa-elcv.ca/). This dataset includes 1,091 individual metabolites and 309 metabolite ratios, generated from plasma samples of 8,299 participants of European ancestry. It is publicly available as fixed content without a separate version number and was downloaded in May 2025.

3. Chronic Rhinosinusitis (CRS) Genome-Wide Association Study (GWAS) Data: Summary statistics for CRS GWAS were sourced from the FinnGen Consortium (Release R12, Phenotype: J10_CHRONICSINUSITIS; URL: https://storage.googleapis.com/finngen-public-data-r12/summary_stats/release/finngen_R12_J10_CHRONSINUSITIS.gz. This dataset is publicly available as fixed content without a separate version number and was downloaded in May 2025. It includes 22,099 CRS cases and 371,520 controls, all from participants of European ancestry.

All datasets can be accessed in accordance with the official usage agreements of the corresponding databases. Typically, users must register or apply for access as specified by each database. No additional restrictions apply to the use of these data for academic research.

## Funding

No external funding was received for this research. All costs were covered by the authors or their affiliated institution.

## Data availability statement

The authors declare that all data are available in repository.

## Declaration of competing interest

The authors declare no competing financial or non-financial interests that could influence the work reported.
