# Supplementary material for: Systematic elucidation of the cross-omics regulatory network in chronic rhinosinusitis: the LAT-IL23R metabolic axis
Source: Braz J Otorhinolaryngol. 2026 Apr 22;92(4):101819. doi: 10.1016/j.bjorl.2026.101819 (PMC13126341; doi:10.1016/j.bjorl.2026.101819)

**BJORL-D-25-00246**

**Material Supplementary**

**Table S1** List of the Identification (ID) for each of the 1400 plasma metabolites.

**Table S2** Causal associations between proteins and chronic rhinosinusitis identified by deCODE pQTL-based Mendelian randomization.

**Table S3** Causal associations between proteins and chronic rhinosinusitis identified by UKB-PPP pQTL-based Mendelian randomization.

**Table S4** Mendelian randomization analysis of causal effects between UKB-PPP pQTL-derived proteins and deCODE pQTL-derived proteins in chronic rhinosinusitis.

**Table S5** Mediation statistics for all tested axes: protein-protein and protein-metabolite pathways in chronic rhinosinusitis.

**Table S6** Mendelian randomization analysis of CRS-associated metabolites and their relationship with IL23R protein.

**Table S7** Detailed information of Single Nucleotide Polymorphisms (SNPs) serving as instrumental variables in Mendelian Randomization (MR) analyses for the LAT-IL23R-amino acid metabolic axis.

**Table S8** Mendelian randomization, sensitivity analysis, and MR-PRESSO results of the LAT-IL23R amino acid axis.

**Table S9** Summary of dataset characteristics and purposes in this study.

**Figure S1**


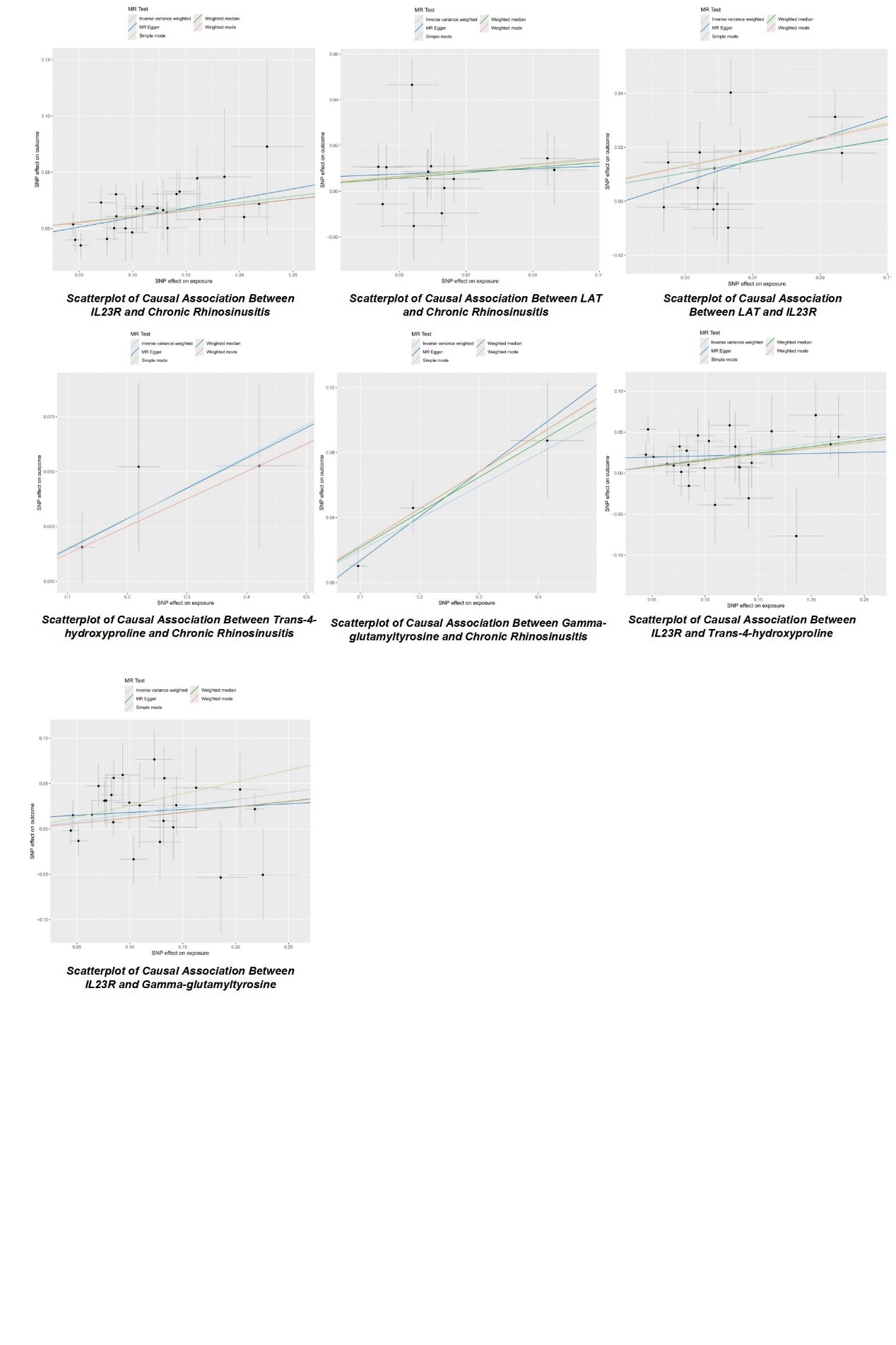


**Figure S2**


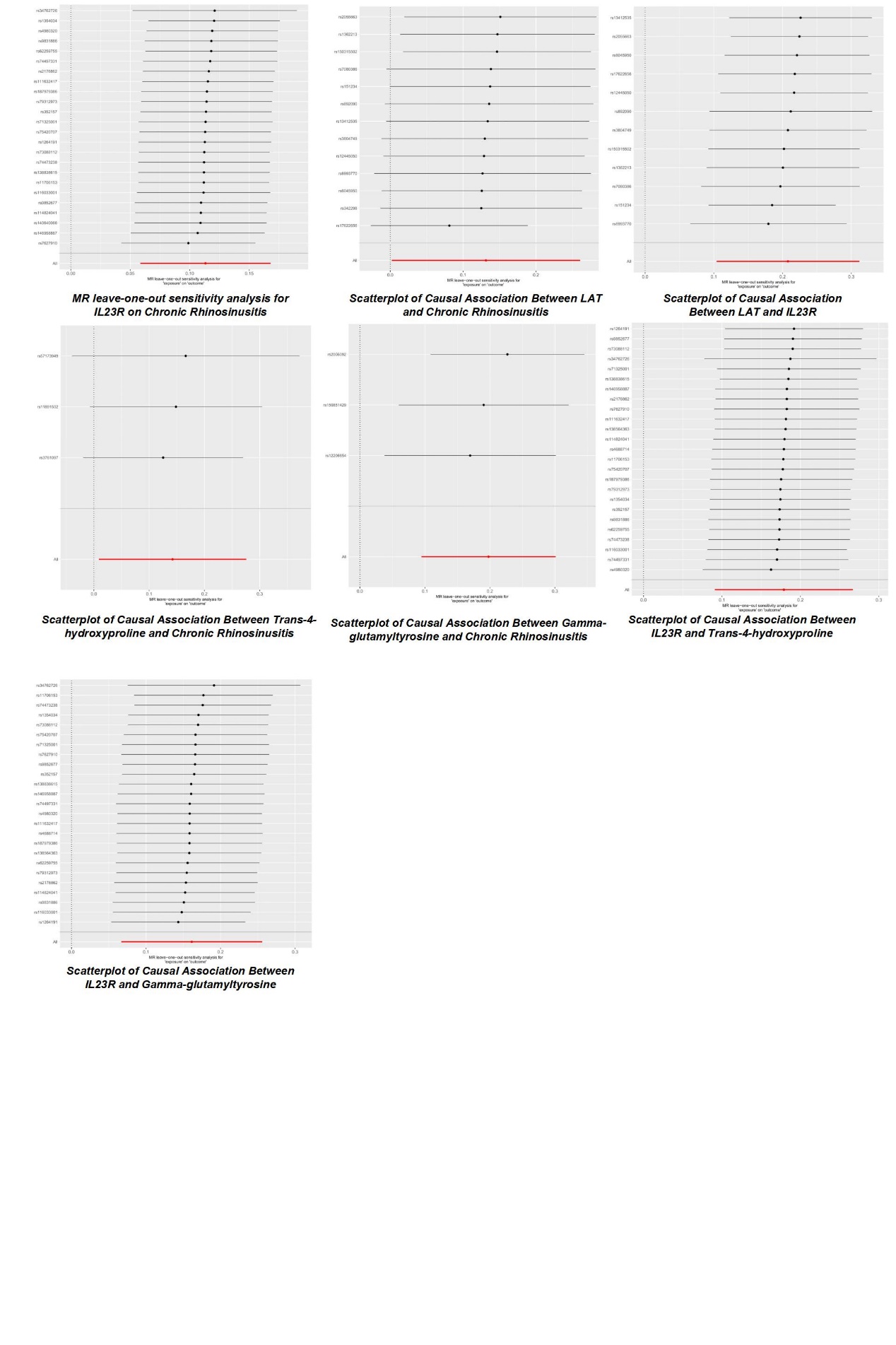


**Figure S3**


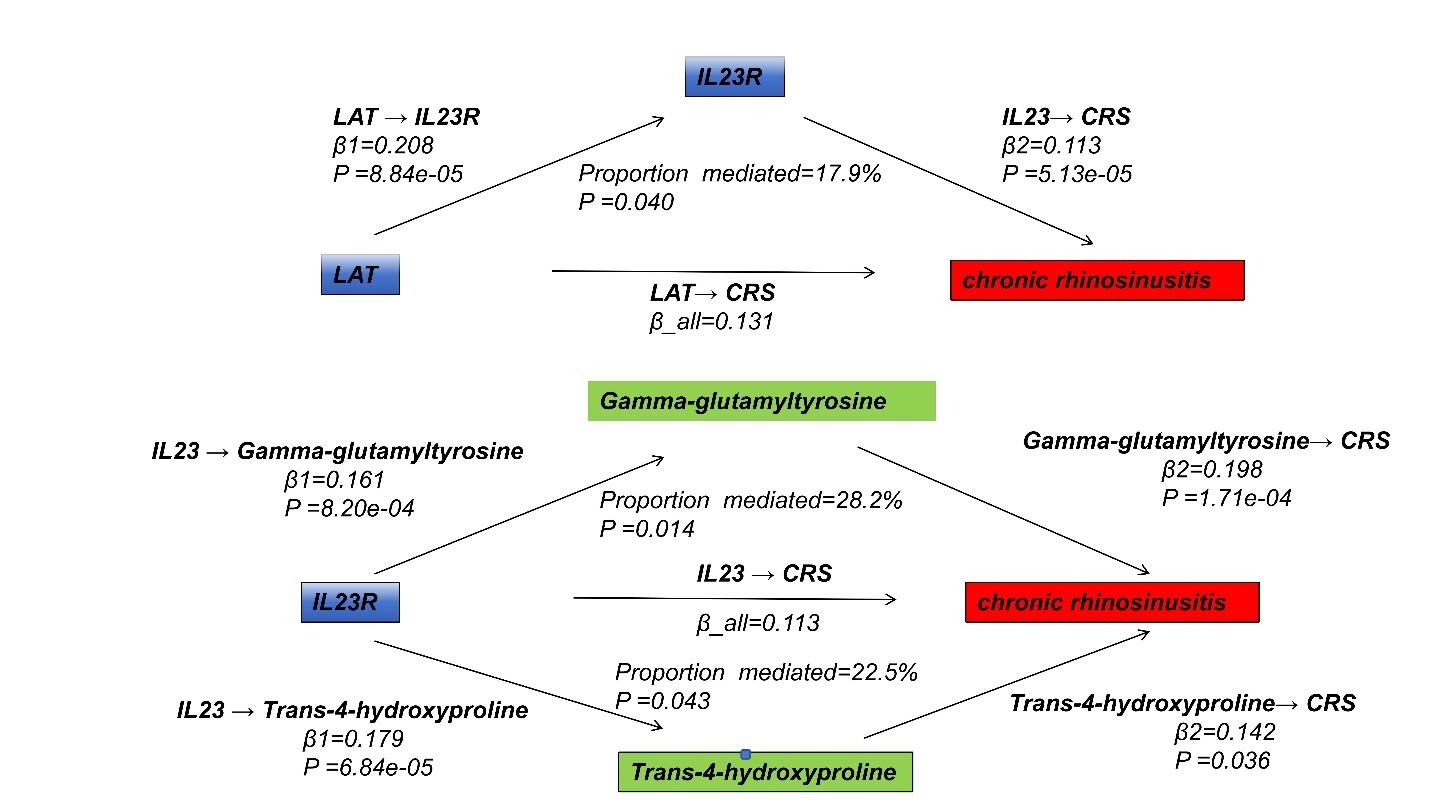

Supplement: Supplementary file 10 [file mmc10.docx]
